# Optimized transmission of multi-path low-latency routing for electricity internet of things based on SDN task distribution

**DOI:** 10.1371/journal.pone.0314253

**Published:** 2025-02-07

**Authors:** Qi Jin

**Affiliations:** University of lllinois Urbana-Champaign Institute, Zhejiang University, Jiaxing, China; Beijing University of Technology, CHINA

## Abstract

With the continuous development of 5G network, how to further improve the routing and transmission efficiency of electric power IoT has become a popular research at present. Facing the current problems of high data transmission delay, low efficiency, and large task volume in the electric power IoT, this research combines software-defined networking to design a multi-path low-latency routing and transmission model under the concept of task allocation. First, the grid data communication network model and network slicing technology in 5G power IoT are introduced. On this basis, considering the data transmission in the core network in the power IoT, a multi-path low-latency routing optimization transmission model based on software-defined network task allocation is designed by combining the software-defined network controller and task allocation concept. The results indicated that the average delay of the designed model is only 15.78ms when the transmission task size is 10KB and 23.38ms when the transmission task size is 50KB. In addition, the designed model was able to achieve a throughput of 298bps in the local area network and the lowest jitter and packet loss in the wide area network, which are 0.13ms and 0.001%. It can be concluded that the constructed multi-path low-latency routing and transmission model can not only provide theoretical guidance for the optimization of data transmission in the power IoT, but also lay the foundation for the in-depth application and development of software-defined networking in the power IoT and other fields.

## 1. Introduction

In the context of the current informationization and networking era, power Internet of things (PIoT), as an important technology to support the development of smart grid and smart city, has become a hot spot for research and application [1,2]. The efficient and stable operation of PIoT plays a crucial role in ensuring the reliability, real-time and security of the power system. However, with the expansion of PIoT scale and the diversification of business requirements, it faces challenges such as high data transmission delay (TD), network congestion and unreasonable resource allocation [3,4]. In this context, how to realize low-latency and high-efficiency transmission of data in PIoT has become an urgent problem to be solved. Software-defined networking (SDN), as an emerging network architecture design concept, can further realize the flexibility and programmability of network control by separating the control plane from the data plane [5,6]. PIoT combined with SDN can dynamically adjust network resources and optimize data transmission path (Trans-P) through centralized network control and management, thus effectively reducing the delay of data transmission and improving the transmission efficiency and reliability of the network [7]. In PIoT system, data transmission faces many practical challenges, such as high data transmission delay, network congestion, and unreasonable resource allocation. These problems seriously affect the reliability, real-time and security of power system. Although SDN technology enables flexibility and programmability of network control by separating the control plane from the data plane, its specific application in PIoT needs to be further optimized. Based on this background, a multi-path low-latency routing transmission optimization model for power Internet of Things based on SDN task allocation is designed, aiming to achieve dynamic adjustment of task allocation and optimization of multipath transmission strategy through this model, so as to reduce data transmission delay and improve network transmission efficiency and stability.

The main contributions of the study are as follows. Firstly, a multi-path low-delay routing optimization transmission model based on SDN task allocation (MPLRM-SDN) is proposed. Combined with network slicing technology, multi-path low-delay data transmission under different task assignments is realized, and network resource utilization and transmission efficiency are improved. Secondly, aiming at problems such as high data transmission delay, network congestion and unreasonable resource allocation in PIoT, this study introduced SDN controller and task allocation mechanism to realize dynamic network resource adjustment and data transmission path optimization through centralized network control and management, which significantly reduced data transmission delay and improved network stability and transmission reliability.

The study is divided into five parts in total, the first part is an introduction to the full study. The second part is an analysis of the related work. The third part is the construction of the multi-path low-latency transmission model. The fourth part is the testing and analysis of the performance of the constructed model. The last part is a summary of the full study.

## 2. Related works

In recent years, SDN has gradually shown significant advantages in the development of intelligent systems for Internet of things (IoT) due to its special centralized architecture. The architecture has been studied by a number of experts as it not only manages sustainable applications efficiently but also reduces communication costs. N. Islam et al. proposed an adaptive routing migration model that aims to optimize data transmission, reduce disconnection time, and enhance the security of network service management and IoT cloud through a three-level security algorithm. Simulation test results revealed that the model outperforms existing techniques in terms of transmission efficiency and security [8]. C. H. Ke et al. proposed a reinforcement learning-based routing method under SDN, naming the method as widest path routing algorithm based on Q-learning. The algorithm was able to find the optimal Trans-P between the source and the destination with maximum band-width through reinforcement learning based on the link band-width processing reward function in the execution environment. Experimental results demonstrated that the proposed algorithm outperforms Dijkstra’s algorithm in finding the widest Trans-P in SDN environments with different band-widths, packet loss rates and background traffic conditions [9]. Traditional routing protocols use limited information in the decision making process, resulting in poor adaptation to traffic variations and limited support for quality of service (QoS) requirements of applications. D. M. Casas-Velasco et al. proposed a novel routing approach in SDN, namely intelligent routing based on reinforcement learning and SDN. Intelligent routing added a knowledge plane to SDN and defined a reinforcement learning based routing algorithm that makes routing decisions by considering link state information. The results of the study showed that intelligent routing outperforms Dijkstra’s algorithm in terms of extension, link throughput, packet loss and delay [10]. In software-defined wireless networks, optimized routing techniques are one of the effective solutions to improve network performance. Common optimization methods include constructing optimal routing metrics using integer linear programming problems, which usually focus on a single routing objective, such as minimizing packet blocking probability, minimizing end-to-end delay, or maximizing network throughput, and it is difficult to consider multiple objectives simultaneously. T. V. T. Duong et al. explored the application of machine learning to the routing control of software-defined wireless networks and proposed a machine learning-based intelligent routing algorithm to enhance network performance and optimize multiple routing objectives. Performance evaluation results revealed that the proposed algorithm significantly improves network performance in terms of packet delivery and network throughput compared to other well-known routing algorithms [11].

With the arrival of the 5G era, low-latency communication routing techniques for smart PIoT have gradually received more and more attention and research. X. Wang et al. considered the most representative features of vehicles and roads to develop a new routing protocol, which is characterized as a low-latency and energy-efficient routing protocol based on network connectivity. The protocol was based on the assumption of uneven distribution of vehicles, analyzed the network as a metric for routing decisions using a non-chiral Poisson process, and made routing decisions under multiple selection criteria through the use of fuzzy logic. Experimental simulation results showed that the proposed protocol provides significant improvement in routing stability and outperforms the classical self-organizing on-demand distance vector routing protocol in several aspects [12]. K. Parane et al. explored field programmable gate array-based network-on-chip design using low-latency routers with prospective bypassing techniques aimed at optimizing area while improving network performance. Compared to ProNoC and CONNECT NoC architectures, the experimental architecture consumed 4.5% and 27.1% less hardware resources, reduced average packet latency by 30% and 15%, and was 1.15x and 1.18x faster than ProNoC and CONNECT NoC architectures, respectively, in terms of performance, demonstrating significant routing stability and multifaceted performance advantages [13]. J. Cheng et al. proposed an interference-coordinated routing scheme for wireless multihop networks, aiming to achieve more transmission concurrency and thus reduce the end-to-end delay. Simulation results confirmed that the designed interference-coordinated routing scheme for wireless multihop networks reduces the end-to-end delay by 9.16% to 73.82% and promotes better transmission concurrency compared to the existing schemes [14]. B. Gokalgandhi et al. proposed the use of reliability and amount of delay as metrics in the routing tree optimization process for Wi-Fi mesh networks. In contrast to existing routing optimization methods, the proposal optimized the data rates of individual mesh links directly based on the underlying channel conditions to meet the reliability and delay requirements of the entire mesh path. In addition, to alleviate the channel contention problem commonly found in Wi-Fi networks, they proposed a multi-channel allocation method. The results indicated that in this method, based on the expected traffic load, this multi-channel allocation method is able to allocate reasonable band-width to individual mesh nodes [15].

In their study of the load balancing opportunity routing problem in wireless sensor networks, M. U. Farooq and colleagues employed SDN to separate the control plane from the sensor node, thereby enabling flexible management. The findings indicated that the protocol significantly enhances the performance of network lifetime, routing efficiency, energy consumption, sending wait time, and repeated packets. Moreover, it offered clear advantages over the benchmark method [16]. R. Ruby et al. studied the routing problem in underwater sensor networks, using SDN to provide a centralized solution. In this study, the routing problem of multimodal underwater sensor networks was described as an optimization problem, considering the flexibility of full duplex and half duplex nodes, and using convex programming relaxation and greedy methods to solve the full duplex and half duplex scenarios respectively. Through Python simulation, it was verified that the proposed global optimal routing scheme is superior to the three existing distributed routing protocols in terms of reliability, delay and energy efficiency [17]. S. Oh et al. proposed an adaptive real-time communication system called RT-SDN. The system used SDN to achieve end to end deadline guarantee. In addition, an efficient routing algorithm was used to configure traffic routes adaptively to ensure band-width. Finally, a new priority assignment scheme was introduced to improve the deadline guarantee, and a feedback loop is used to realize the cooperative work of routing and scheduling. The results showed that the RT-SDN prototype validates its ability to provide end-to-end deadline assurance in real networks and significantly improves schedulability [18].

In conclusion, these methods demonstrate efficacy in their respective operational contexts. However, they are constrained by limitations in the face of challenges such as high latency, network congestion, and the allocation of resources in an unreasonable manner within the PIoT. This study presents a multi-path low-latency routing optimization transmission model that combines SDN dynamic resource management and network slicing technology. The objective is to address the challenges of high data TD, network congestion, and unreasonable resource allocation in the PIoT environment.

## 3. PIoT routing optimization transmission study

In order to improve the RT effect of 5G-PIoT, this research firstly introduces the communication networks (CN) model and network slicing technique for grid data for PIoT. Based on this, the core network transmission (CNT) model is used as the research object to further focus on the optimization of the core network band-width and data transmission volume, and a multi-path low-latency transmission model is proposed, aiming to complete the multi-tasking allocation by using SDN in order to further improve the efficiency of network slicing, and ultimately realize the multi-path low-latency RT.

### 3.1. Communication networks modeling and network slicing techniques for PIoT-oriented grid data

**Fig 1:**
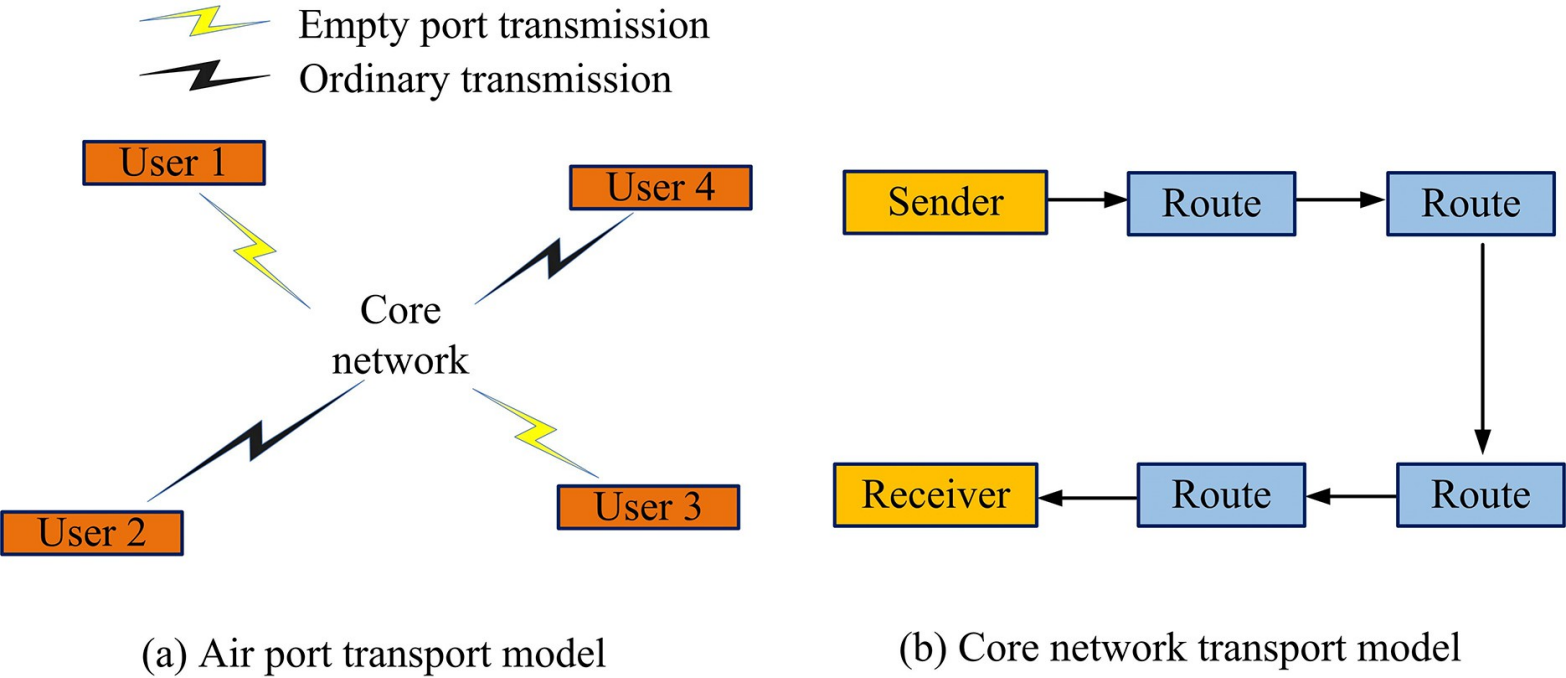
Structure of the lip-service transmission model and core network transmission model.

PIoT refers to the introduction of IoT technology into the traditional power system, which realizes real-time monitoring, management, and optimization of the power system by installing sensing devices (smart sensors, smart meters) in each link of power generation, transmission, distribution, and consumption and by using communication technologies and cloud computing platforms. In the 5G era that has arrived, the continuous optimization of PIoT aims to further improve the operational efficiency and reliability of the power system, reduce the operation and maintenance costs, and at the same time enhance the quality of power services and user experience. The CN of grid data is an important component of PIoT, which is mainly responsible for transmitting and processing data information in the power grid. There are two main models related to the CN of grid data, which are the air-port transmission (APT) model and the CNT model, and the structure of the two models is shown in Fig 1.

In Fig 1, Fig 1A and 1B show the structure of the APT model and the CNT model, respectively. In the APT model, the end-to-end delay of PIoT data transmission is divided into three parts, which are the air-port TD at the sending end, the air-port TD at the receiving end, and the TD within the core network. In the CNT model, the delay in the core network part consists of TD, queuing delay, and processing delay [19,20].

Assuming that the null port TD at the transmitter is *t*_*in*_, the expression for *t*_*in*_ is obtained as shown in Eq (1).


$$t_{in}=V{\left[B_{in}\;\log_{2}(1+\frac{P_{s}{\left|h_{s,BS}\right|}^{2}}{N_{0}B_{in}})\right]}^{-1}$$ (1)

In Eq (1), *V* denotes the size of the packet sent by the service and *s* denotes the service sending terminal. *B*_*in*_ denotes the band-width of the radio access network on the *s* side, and *P*_*s*_ denotes the transmission power (TP) of the *s*. *h*_*s*,*BS*_ denotes the channel gain from *s* to *BS*, and *BS* denotes the base station. *N*_0_ denotes the power spectral density of the noise.

Assuming that the air-port TD at the receiver is *t*_*out*_, the expression for *t*_*out*_ is obtained as shown in Eq (2).


$$t_{out}=V{\left[B_{out}\;\log_{2}(1+\frac{P_{BS}{\left|h_{BS,r}\right|}^{2}}{N_{0}B_{out}})\right]}^{-1}$$ (2)

In Eq (2), *r* denotes the service receiving terminal and *B*_*out*_ denotes the radio access network band-width on the side of *r*. *P*_*BS*_ denotes the TP of *BS* and *h*_*BS*,*r*_ denotes the channel gain from *BS* to *r*.

In the CNT model, the TD is assumed to be *t*_*t*_. The formula for *t*_*t*_ is shown in Eq (3).


$$t_{t}=\frac{V}{R}$$ (3)

In Eq (3), *R* denotes the transmission rate (TR), and its specific formula is shown in Eq (4).


$$R=B_{core}\;\log_{2}(1+\frac{P_{w}}{N_{0}B_{core}})$$ (4)

In Eq (4), *B*_*core*_ denotes the band-width of the core network, *w* denotes the routing node, and *P*_*w*_ denotes the transmit power of *w*.

**Fig 2:**
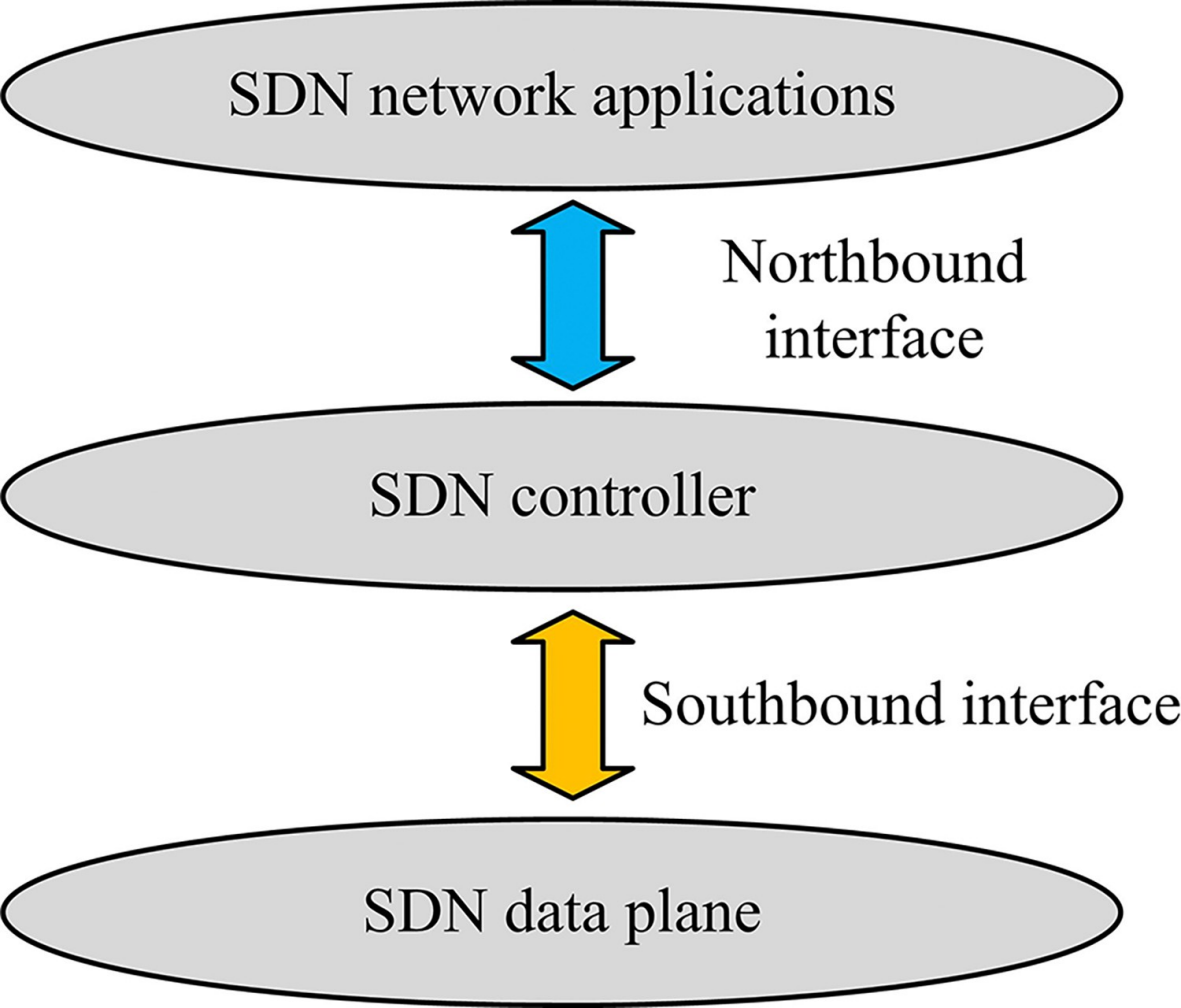
SDN architecture diagram.

SDN is a network architecture whose core idea is to control the behavior of the network through software applications rather than relying on traditional physical hardware devices [21,22]. The main feature of SDN is that it separates the network control layer from the data forwarding layer, making network management more flexible and centralized. In SDN, network administrators are allowed to programmatically control network traffic dynamically and centrally without physically touching individual switches. Compared to other network architectures, SDN not only improves network resource utilization and simplifies network configuration and management, but also supports more flexible data traffic management and innovative network management strategies. Although SDN technology is not yet fully adapted to IoT and constrained devices, it can be effectively applied to PIoT environments in the following ways. First, to accommodate resource-constrained PIoT devices, lightweight SDN controllers can be designed and deployed that operate efficiently on resource-constrained devices. Secondly, the distributed SDN architecture is adopted in PIoT, which enables the distribution of control functions to multiple nodes, reduces the risk of a single point of failure, and improves the reliability and scalability of the system. Finally, SDN can be combined with edge computing to perform data processing and decision-making in close proximity to the data source, thereby reducing the latency and band-width requirements of data transmission and improving the overall network performance. The aforementioned enhancements facilitate a more optimal integration of SDN technology within the context of the PIoT environment. This integration enables the delivery of low-latency, high reliability, and flexible network services. The architecture diagram of SDN is shown in Fig 2.

The architecture of SDN is illustrated in Fig 2, which consists of five main components: the SDN network application layer (AL), the SDN controller, the SDN data plane, the southbound interface, and the northbound interface [23]. The AL is responsible for implementing network services and contains the necessary transport protocols and QoS metrics. Service requests are processed by the AL, and the relevant protocols and resource configuration instructions are passed from the AL to the SDN controller via the northbound interface. The northbound interface allows the exchange of information between the AL and the controller, passing service resource control commands through a unified protocol. The SDN controller centrally manages the network data, not only providing a service-oriented programming platform, but also responsible for path provisioning and status monitoring to ensure the implementation of applications. The communication between the controller and the data plane is carried out through the southbound interface, which mainly uses the Openflow protocol to realize the instruction issuance and the unified operation of network devices. The data plane is responsible for executing the commands issued by the controller, completing the realization of network functions, and regularly feeding back the network status to the controller to ensure the global management of the network.

**Fig 3:**
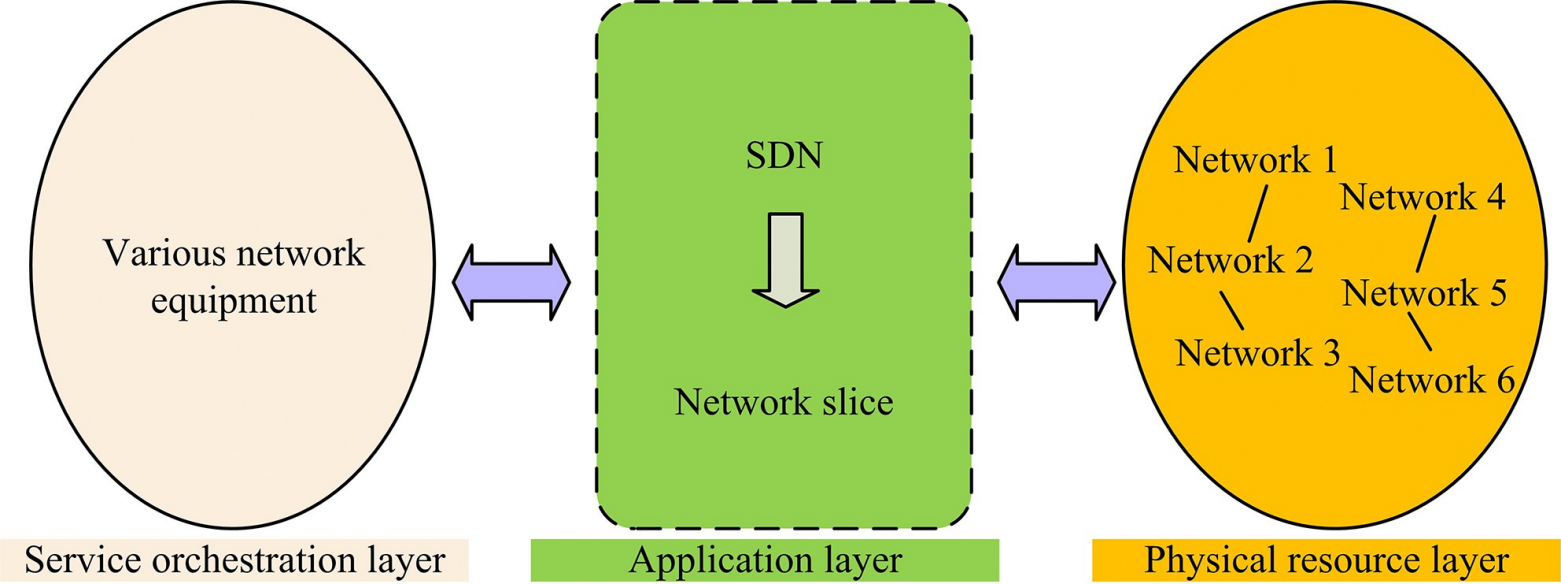
Deployment architecture of network slicing.

Network slicing technology significantly enhances the reliability and security of data transmission by replacing the traditional centralized data processing approach with a customized approach oriented to services and services [24,25]. This technique utilizes QoS metrics to construct network slicing, which not only ensures efficient allocation of the underlying physical resources, but also meets the latency, rate, and reliability requirements of specific services. Meanwhile, service function chaining (SFC) is utilized to achieve logical isolation between different services and enhance transmission security. In addition, network slicing can isolate faults and prevent problems in one slice from affecting other slices, ensuring the overall stability of the network. Therefore, service-oriented network slicing is a key advantage in 5G communications [26]. The core of realizing network slicing technology lies in the decoupling feature of SDN. This feature allows the network to be customized and configured according to user needs, which not only simplifies network deployment but also improves service processing efficiency. In the 5G environment, this flexible resource management and slicing construction is the key to providing differentiated network services. Operators can flexibly deploy the underlying resources according to the type of service and quality requirements to achieve dynamic adjustment of the network structure, so as to meet the demand for different services in terms of latency, band-width, power, and packet loss rate and other indicators. The deployment structure of network slicing is shown in Fig 3.

**Fig 4:**
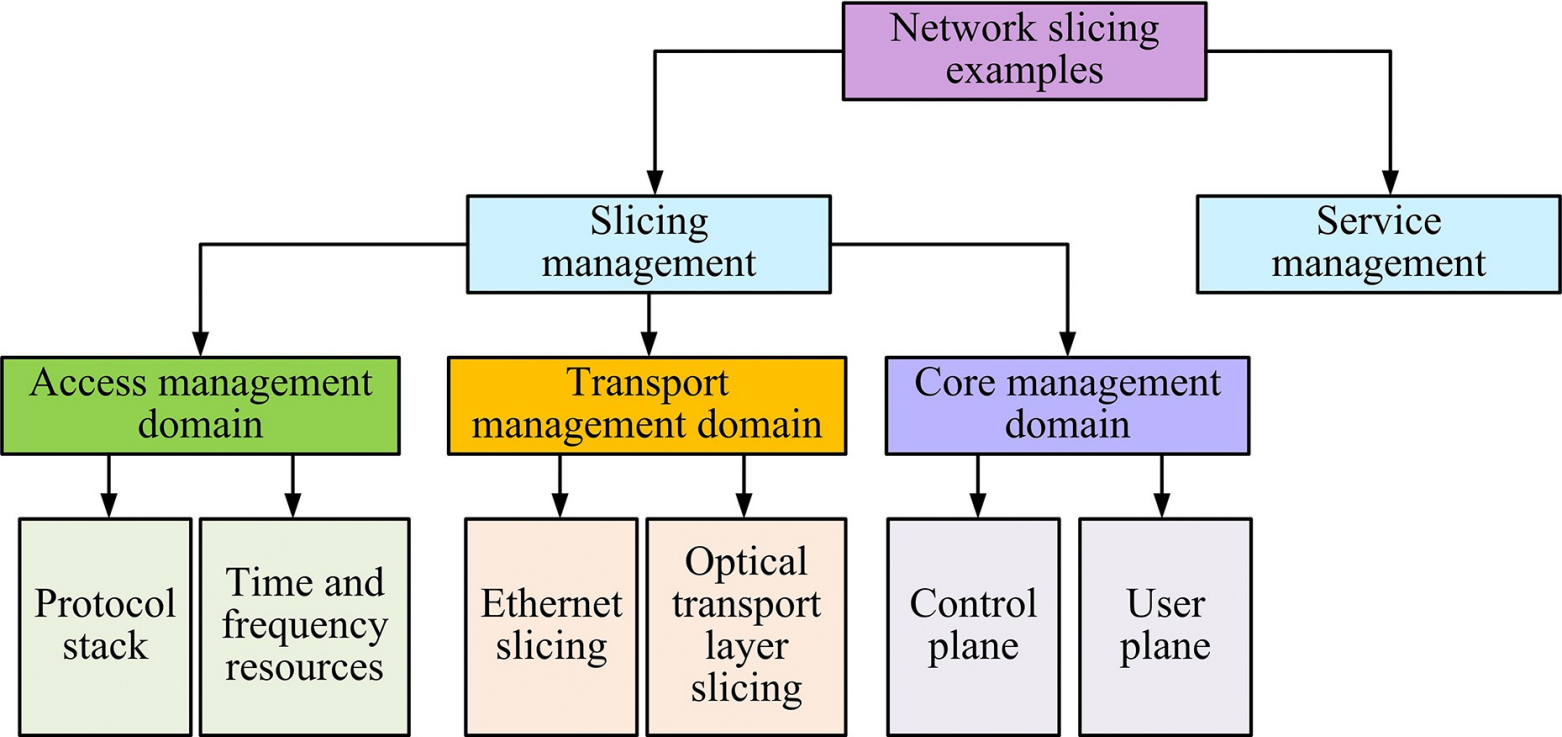
Flowchart of the management collaboration between the various parts of the network slice.

Fig 3 shows the deployment structure of network slicing, which can be mainly divided into AL, service orchestration layer, and physical resource layer (PRL). First, the requirements of information modeling metrics, virtual network functions, and network resources needed at the AL will be formulated by the service provider and stored at the operator side. Upon receiving a network application request, the operator utilizes the SDN controller to schedule the required network resources by matching the service requirements with the corresponding network slice (NS) in order to configure the network functions, activate the services, and generate NS instances. Next, the SDN controller is responsible for centralized scheduling and real-time monitoring of the global network to adapt to changes in service demand and reconfigure the underlying resources in the event of failure to ensure stable operation of the NS. Upon service completion or demand termination, the SDN controller releases the occupied resources. The management collaboration between the various parts of the NS is shown in Fig 4.

**Fig 5:**
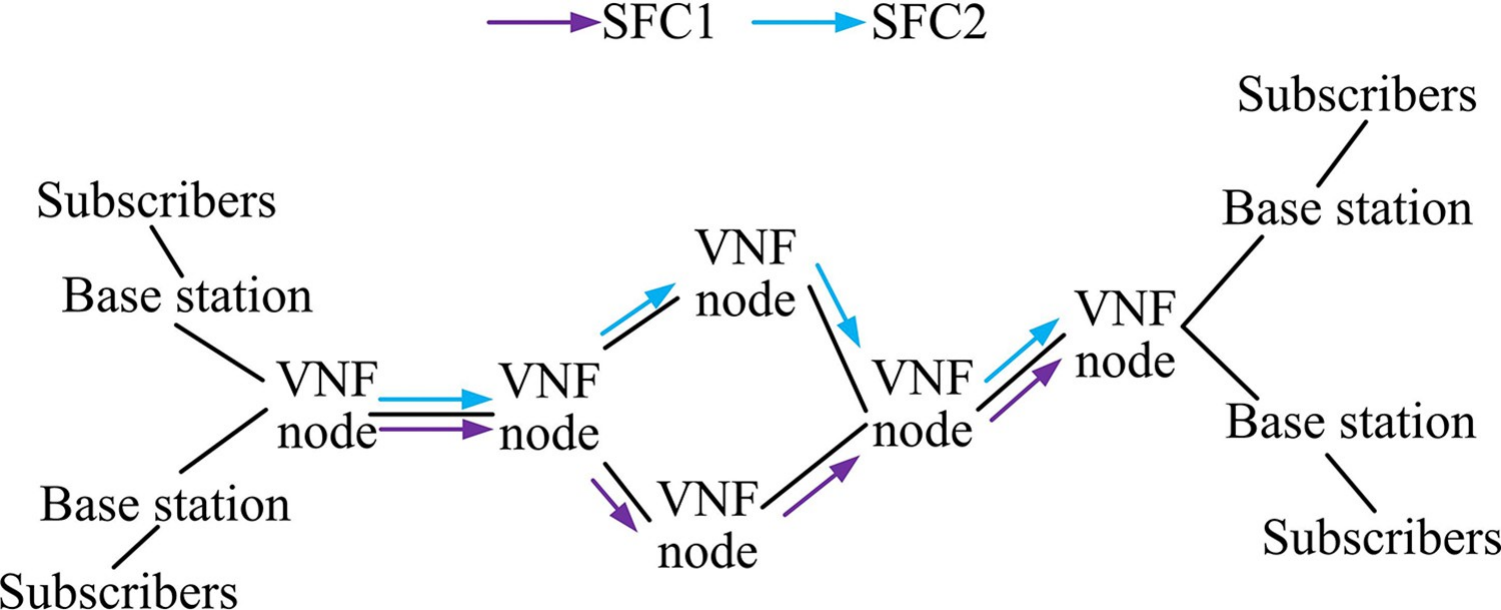
Deployment diagram of the function chain to realize network slicing.

The management and collaboration flowchart between various parts of the network slicing is given in Fig 4. The realization and management of NSs require corresponding collaboration among various parts of the network. Moreover, the management and collaboration process involves the service and application management of service providers, the invocation of network resources, the instantiation of slices, and the differentiation of different service identifiers. At the terminal level, user devices access the corresponding slices according to the NS identity. The radio access network is responsible for resource allocation and isolation, the transmission network realizes the logical isolation of different slices, and the core network supports the separation of control and bearer, realizes the logical isolation of services and customizes the slicing function to enhance the robustness of the network. The realization of network slicing is shown in Fig 5.

The deployment of SFC for realizing network slicing is given in Fig 5. The key to achieving network slicing lies in the deployment of SFCs, in which network function virtualization (NFV) technology abstracts physical resources into virtual resources, and the SFCs formed by different NFVs are called through SDN controllers. Each link must satisfy a specific QoS requirement to ensure the implementation of business functions and logical isolation. The deployment of SFCs affects the quality of data transmission, including latency and reliability. appropriate deployment of SFCs can meet different business requirements and optimize the performance of network slicing with limited resources.

### 3.2. Design of multi-path low-latency routing-optimized transmission model based on SDN task assignment

**Fig 6:**
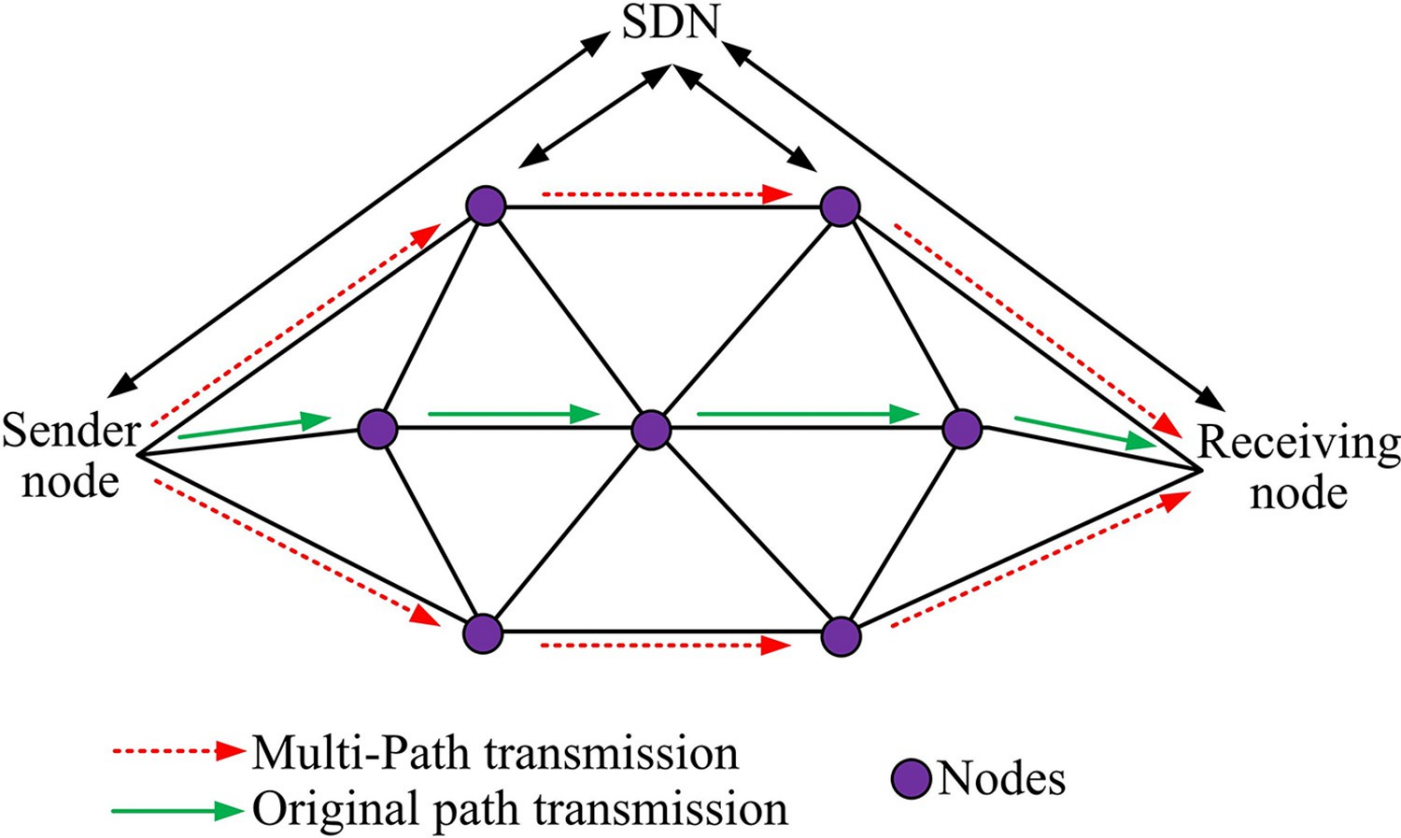
Deployment architecture diagram of MPLLRM-SDN model.

For the data transmission scenario in PIoT, section 3.1 firstly introduces two data transmission models, and then introduces SDN and network slicing techniques. In order to further consider the data transmission scenario in the core network in PIoT and reduce its TD, the study further proposes an MPLLRM-SDN based on 2.1. In a PIoT environment, data traffic is characterized by high dynamism. The separation of the control plane from the data plane enables the implementation of centralized management and dynamic adjustment of network resources. This allows for the selection of the optimal path according to the real-time network conditions, thereby reducing the delay and congestion of data transmission. Network slicing technology enables the creation of independent virtual networks based on specific application requirements, thus providing customized QoS assurance. The task assignment mechanism proposed in this study can be intelligently assigned to multiple Trans-Ps according to the priority of tasks and the amount of data, further improving transmission efficiency and reliability. The deployment structure of MPLLRM-SDN model is shown in Fig 6.

The deployment architecture of the MPLLRM-SDN model is given in Fig 6. In the PIoT scenario, the application architecture of the multi-path low-latency routing optimization scheme based on task assignment is used, which consists of the SDN control layer and the PRL. NFV nodes are utilized to act as routing nodes responsible for processing and forwarding transmission services [27,28]. Considering the grid differential protection scenario, the task data is divided into multiple packets and forwarded to the target endpoints among the routing nodes of the network. MPT enables packets to be transmitted through different routes and converged at the end point. The PRL covers the Trans-Ps and links. The SDN controller is responsible for scheduling these physical resources and setting up the Trans-Ps. It is able to dynamically monitor the service speed, queue size, and band-width of the links for each Trans-P in order to satisfy the different quality requirements of the transmission tasks in terms of band-width, latency, and rate. At the data transmission level, routing nodes sequentially forward their respective transmission tasks based on the controller’s instructions. After the multi-path low-latency routing optimization scheme based on task allocation reaches the core network, the SDN controller will actively obtain the current routing situation, at this time, the total TD needs to be used as a weight to find the optimal Trans-Ps and sub-optimal Trans-Ps by Dijstra’s algorithm.

The conventional single-path transmission method is susceptible to significant delays in the context of a substantial number of data transmission tasks, which ultimately results in a reduction in data transmission efficiency. The MPLRM-SDN proposed in this study can effectively reduce the data TD by using multiple Trans-Ps and combining the task allocation mechanism. At the same time, the existing methods often reduce the transmission efficiency due to network congestion in the process of data transmission. By introducing SDN controller and network slicing technology, this study realizes the dynamic adjustment and optimization of network resources, and significantly alleviates the problem of network congestion. It is challenging to ensure the stability and reliability of data transmission in the context of a complex PIoT environment, particularly when relying on existing methods. However, the model presented in this study significantly enhances the stability and reliability of data transmission through the centralized control and dynamic adjustment capabilities of SDN.

Assuming that the optimal Trans-P and the sub-optimal Trans-P under the current transmission task volume are denoted as *I* and *J*, respectively, the formula for obtaining the TD of the transmission task packets in the core network is shown in Eq (5) [29,30].


$$t=\sum_{k=1}^{K}\frac{V}{R_{k}}$$ (5)

In Eq (5), *t* denotes the TD of the transmission task packet in the core network, and *K* denotes the CNT link. *R*_*k*_ denotes the TR of the service in the *k* segment on *K*. In the context of MPT, the original packet is assigned to the optimal Trans-P and the sub-optimal Trans-P, therefore, the TD of the packet in the optimal Trans-P is obtained as shown in Eq (6).


$$t^{1}=\sum_{i=1}^{I}\frac{V_{1}}{R_{i}}$$ (6)

In Eq (6), *t*^1^ denotes the TD in the optimal Trans-P, and *V*_1_ denotes the packet size transmitted on the optimal transmission link. *I* denotes the optimal transmission link of the core network, and *R*_*i*_ denotes the TR of the *i* segment on *I*. The specific formula for *R*_*i*_ is shown in Eq (7).


$$R_{i}=B_{core}^{1}\;\log_{2}(1+\frac{P}{NB_{core}^{1}})$$ (7)

In Eq (7), $B_{core}^{1}$ denotes the CNT band-width of *V*_1_ and *N* denotes the noise power spectral density of the core network. *P* denotes the TP of the core network by the node. The TD of the packet in the sub-optimal Trans-P is shown in Eq (8).


$$t^{2}=\sum_{j=1}^{J}\frac{V_{2}}{R_{j}}$$ (8)

In Eq (8), *t*^2^ denotes the TD in the sub-optimal Trans-P and *V*_2_ denotes the packet size transmitted on the sub-optimal transmission link. *J* denotes the sub-optimal transmission link in the core network, and *R*_*j*_ denotes the TR of the *j* segment on *J*. The formula for *R*_*j*_ is shown in Eq (9).


$$R_{j}=B_{core}^{2}\;\log_{2}(1+\frac{P}{NB_{core}^{2}})$$ (9)

In Eq (9), $B_{core}^{2}$ denotes the CNT band-width of *V*_2_ A. Considering that the task packets of the optimal Trans-P and the sub-optimal Trans-P are sent out at the same time, the whole transmission process is terminated when the packets on both links reach the receiving end of the core network. Finally, the total delay of multi-path task transmission is obtained as shown in Eq (10).


$$t_{total}=\max(t^{1},t^{2})=\max(\sum_{i=1}^{I}\frac{V_{1}}{R_{i}},\sum_{j=1}^{J}\frac{V_{2}}{R_{j}})$$ (10)

In Eq (10), *t*_*total*_ denotes the total delay of multi-path task transmission.

**Fig 7:**
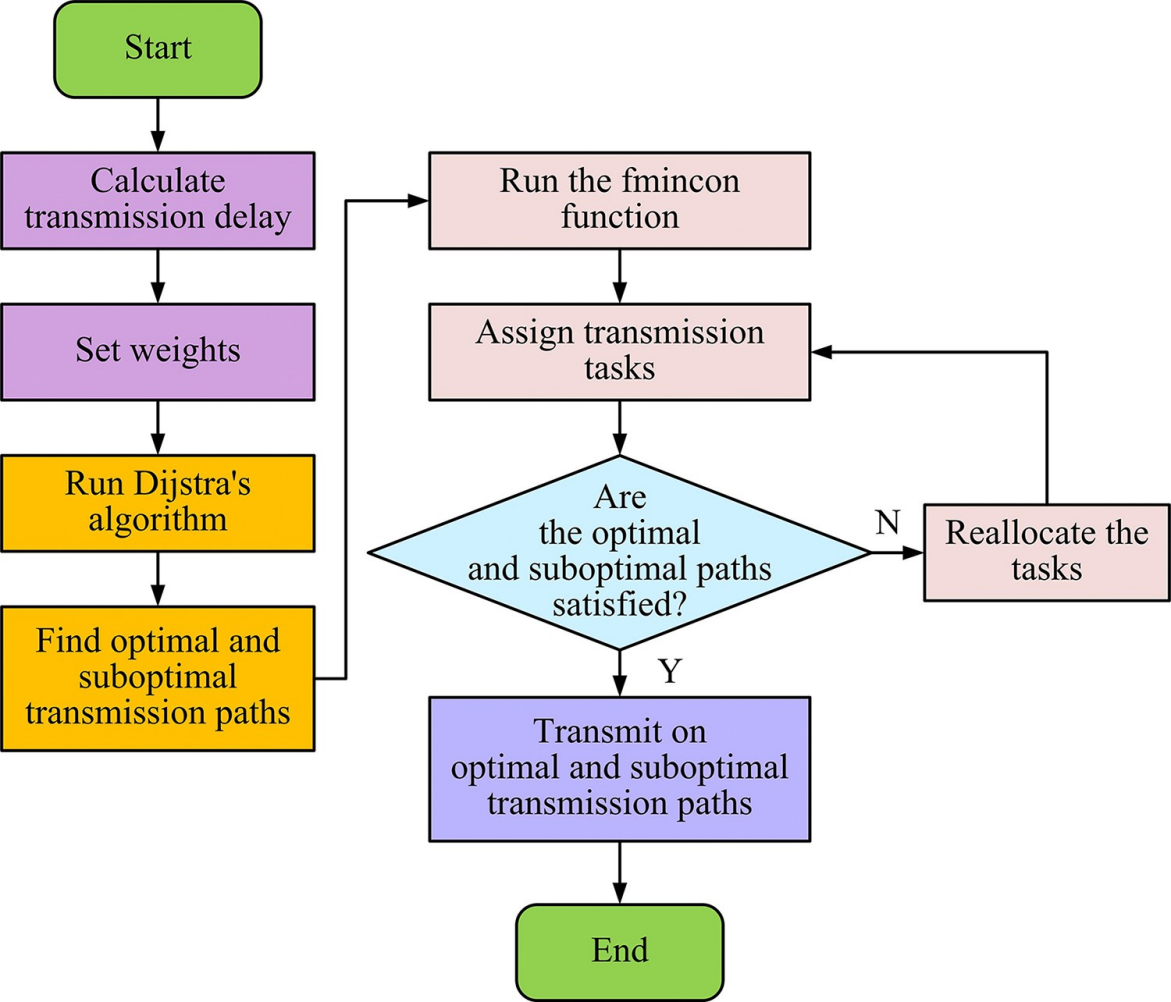
Operational flowchart of the MPLLRM-SDN model.

In the single path transmission model, a transmission route is usually selected by a polling algorithm or an optimal QoS algorithm when the task arrives at the core network [31–33]. This path selection depends on QoS parameters such as TD, band-width, and packet loss rate. However, with the increase in transmission QoS requirements, the traditional single-path transmission approach is no longer able to meet the growing service demands. Single-path transmission may lead to increased latency in data transmission and routing node processing as well as queuing delays under increased load. Once the queuing length of the routing node exceeds the set threshold, the execution of the packet will experience a drop operation, thus increasing the packet loss rate. In addition, the network utilization of this single-path transmission method is not high, and the unused routing resources are regarded as wasteful. Therefore, this research proposes an MPT strategy and builds the MPLLRM-SDN model. In the MPLLRM-SDN model, the utilization of network resources is improved by allocating the task volume to the optimal and sub-optimal transmission links in the network to reduce the TD. By utilizing more routing nodes in the network, the MPT not only reduces the processing burden and the risk of data loss at each node, but also increases the reliability of task transmission. When constructing MPLRM-SDN model, it is necessary to consider a series of constraints to ensure the validity and reliability of the model. First, the total number of transmission tasks must be reasonably distributed between the optimal path and the suboptimal path. This means that the sum of the number of transfer tasks on these two paths must equal the total number of transfer tasks. Second, the distribution of the number of transmission tasks on each path must be non-negative. This is to ensure that the number of transmission tasks on each path is not less than zero to avoid negative transmission. Finally, in the core network, the choice of transmission path should be based on the real-time network conditions, considering the transmission bandwidth, transmission power and network noise and other factors to minimize the total transmission delay. The operation flowchart of MPLLRM-SDN model is shown in Fig 7.

As shown in Fig 7, the study adopts a multi-path task transmission method, dividing the original task volume and scheduling it onto the optimal and sub-optimal transmission paths in the network to reduce transmission delay. At the same time, multi-path transmission makes use of more routing nodes in the network, improving the network’s resource utilization rate. The smaller task volume reduces the processing volume of each routing node and the probability of data packet loss, also enhancing the reliability of task transmission. First, the final TD value is calculated according to the aforementioned Formulas (5) to (10), and this value is used as the weight value. The Dijkstra algorithm is then used with transmission delay as the weight to determine the optimal and sub-optimal transmission paths. Next, the fmincon function is used to allocate the transmission task volume according to these paths. Then, data transmission is carried out on the optimal and sub-optimal transmission paths based on the reallocated task volume. Finally, the process ends. The SDN controller obtains the path bandwidth of the selected path for task allocation in the multi-path low-latency routing optimization strategy. The optimization problem proposed in the study, based on the task allocation multi-path low-latency routing optimization strategy, is represented by the data model, as shown in Formula (11).


$$\{\begin{array}{c} \left[V_{1},V_{2}]=\arg\;\min\;\max(t^{1},t^{2})=\arg\;\min\;\max(\sum_{i=1}^{I}\frac{V_{1}}{R_{i}},\sum_{j=1}^{J}\frac{V_{2}}{R_{j}}\right)\\ C1:V_{1},V_{2}\geq 0\\ C2:V_{1}+V_{2}=V_{total} \end{array}$$ (11)

The mathematical expression of the MPLRM-SDN model and its constraints are given in Eq (11). Where, both *C*1 and *C*2 denote the constraints of the RT optimization strategy. the value of *C*1 is to ensure that the size of the packets transmitted on both the optimal and sub-optimal transmission links is not less than 0, and the value of *C*2 is to keep the sum of the MPT tasks unchanged so as to ensure the reliability of the MPTs. the value of *V*_*total*_ denotes the sum of the MPT tasks. According to Eq (11), it is necessary to compare the sizes of *t*^1^ and *t*^2^ so as to discuss the optimal *V*_1_ and *V*_2_ in two cases. When *t*^1^≥*t*^2^, Eq (10) becomes Eq (12) which is shown below.


$$t_{total}=\sum_{i=1}^{I}\frac{V_{1}}{R_{i}}$$ (12)

A first order partial derivation of *V*_1_ of Eq (12) yields Eq (13) as follows.


$$\frac{\partial t_{total}}{\partial V_{1}}=\sum_{i=1}^{I}\frac{1}{R_{i}}$$ (13)

The second-order partial derivation of *V*_1_ in (13) is performed, which in turn yields a second-order partial derivation of *V*_1_ of 0. At this point the optimization function in this case is Hessian matrix semi-positive definite. When *t*^1^<*t*^2^, Eq (10) becomes Eq (14), which is shown below.


$$t_{total}=\sum_{j=1}^{J}\frac{V_{2}}{R_{j}}$$ (14)

A first order partial derivation of *V*_2_ of Eq (14) yields Eq (15) as follows.


$$\frac{\partial t_{total}}{\partial V_{2}}=\sum_{j=1}^{J}\frac{1}{R_{j}}$$ (15)

The second-order derivation of *V*_2_ in (15) is found to be 0. The second-order derivation of *V*_2_ is also found to be 0. At this time, the optimization function is also semi-positive definite for the Hessian matrix. Combining Eq (11)–(15), the optimal *V*_2_ and *V*_2_ are obtained by the joint solution, and the multi-path low-latency routing optimization transmission task is finally completed.

## 4. Analysis of PIoT routing optimization results

In order to prove the effectiveness of the multi-path low-latency transmission strategy proposed in this study, the study introduces the single-path output model and the equalized task transmission model for comparison, and simulation experiments are carried out by selecting the average latency, throughput, jitter, and packet loss rate. Finally, the practical application effects of different transmission models are also analyzed, and it is found that the designed MPLLRM-SDN model has lower TD and faster response time.

### 4.1. MPLLRM-SDN model performance testing

In order to demonstrate the performance of the MPLLRM-SDN model, the study builds a simulation environment using Matlab software. Considering that all the service data are transmitted on the routing nodes of the core network, the transmission order is arranged according to the SDN controller, and the simulation environment is set up as shown in Table 1.

**Table 1:** Simulation environment configuration table.

Experimental equipment	Value
CPU	Intel(R) Core i7-9700K @ 3.6GHz
RAM	64.0GB RAM
Graphics card	NVIDIA GeForce GTX 1660
Number of core network routes	0~10pcs
Transmission links	15 bars
Transmission task size	1KB~50KB
Transmission rate	1Mbps~20Mbps

**Fig 8:**
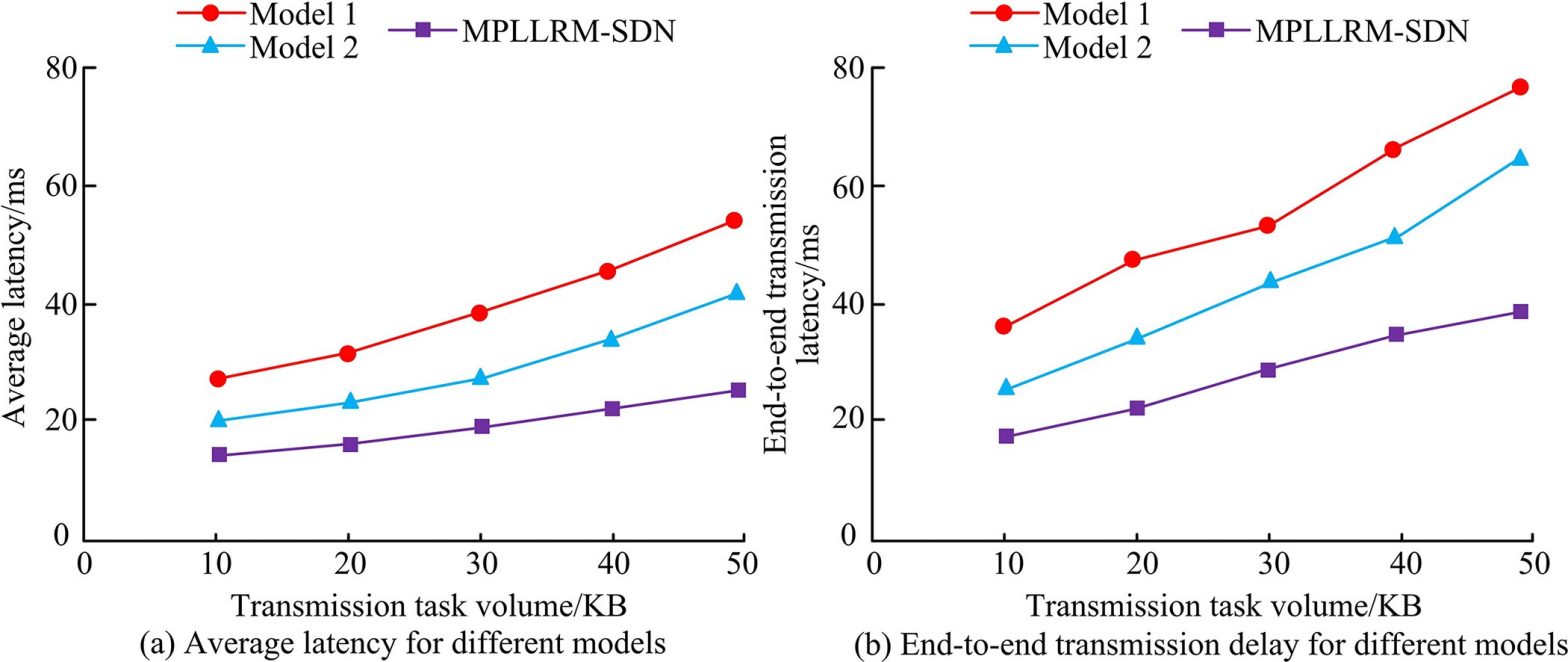
Average delay and end-to-end transmission delay for the three models.

The environment configuration parameters for this experiment are given in Table 1. The number of core network consists in the simulation environment is set to 10, the number of transmission links is set to 15, the transmission task sizes are all in the range of 1KB~50KB, and the TRs of the links are all in the range of 1Mbps~20Mbps. The single-path task transmission model (Model 1) and the equalized task transmission model (Model 2) are introduced for comparison, and the average delay and end-to-end delay of the three models under the same simulation conditions are obtained as shown in Fig 8.

**Fig 9:**
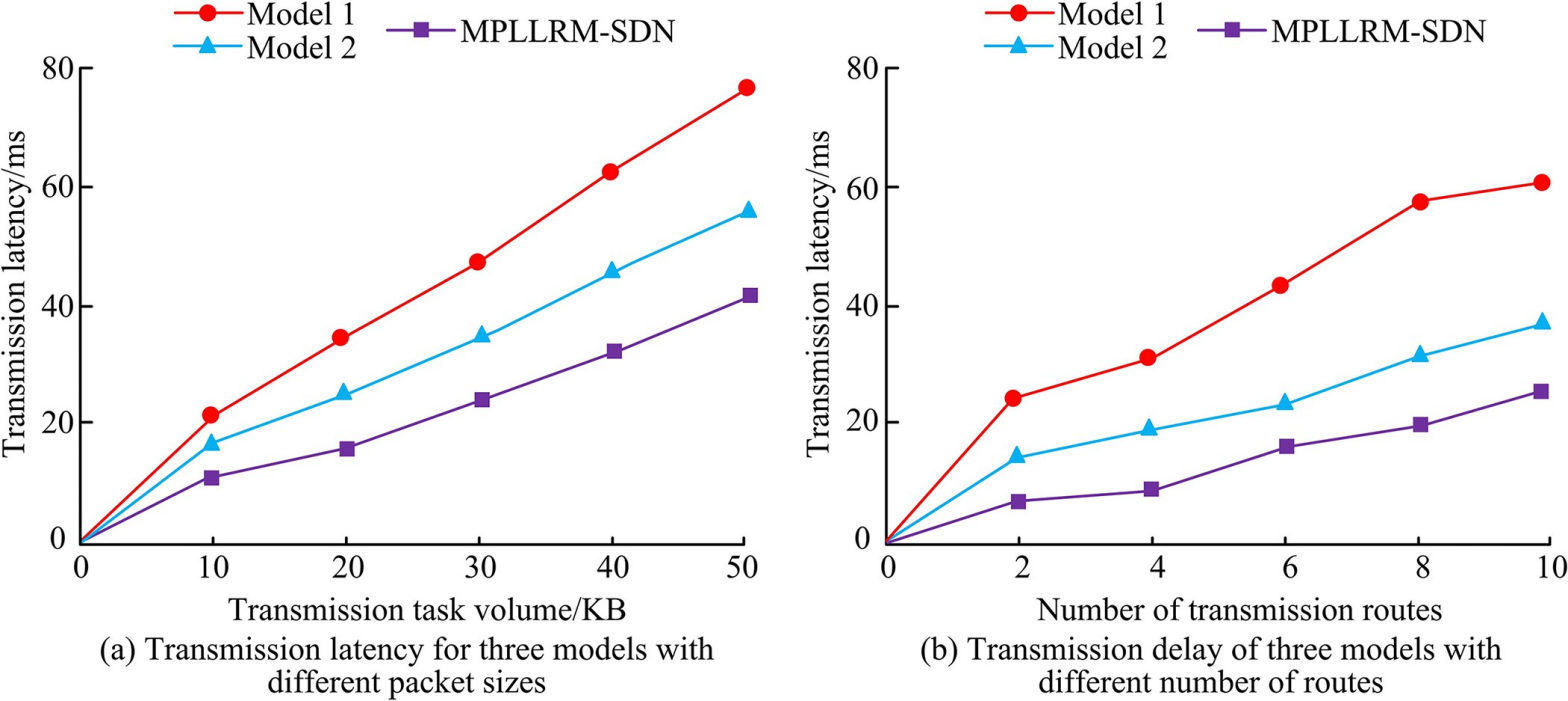
Service transmission delay values for the three models under different conditions.

Fig 8A and 8B show the average delay and end-to-end TD of the three models under different transmission task volumes, respectively. In Fig 8A, the average delay of model 1, model 2, and model MPLLRM-SDN increases from 26.32ms, 19.96ms, and 15.78ms to 53.27ms, 40.15ms, and 23.38ms, respectively, when the transmission task volume increases from 10KB to 50KB. Among them, MPLLRM-SDN has the smallest increase, and its average delay value does not change much with the fluctuation of transmission task volume. In Fig 8B, the end-to-end TD values of the three models increase when the transmission task volume keeps increasing. When the transmission task volume is 50KB, model 1 has the largest end-to-end TD of 78.69ms, and model MPLLRM-SDN has the smallest end-to-end TD of 36.59ms. The service TD values of the three models are tested for different transmission task volumes and different numbers of transmission routes, and the comparative results of the different models are shown in Fig 9.

The changes in service TD for the three models with different transmission task volumes and different numbers of transmission routes are shown in Fig 9A and 9B, respectively. Combining Fig 9A and 9B, it can be observed that the delay values of all the three models increase with the increase of the transmission task volume and the number of transmission routes, and the MPLLRM-SDN model designed in this research has the smallest delay variation compared to Models 1 and 2, while the variation of Model 1 is the largest. The maximum service TD values for Model 1, Model 2, and MPLLRM-SDN are 79.02ms, 54.29ms, and 41.02ms, respectively, when the number of transmission tasks is 50 KB. When the number of transmission routes is 10, the maximum service TD values are 63.71ms, 37.69ms, and 22.58ms for Model 1, Model 2, and MPLLRM-SDN, respectively, 22.58ms. three different network types, local area network (LAN), metropolitan area network (MAN) and wide area network (WAN) are set and the throughput, jitter value and packet loss rate of the three models under the three network types are obtained as shown in Table 2.

**Table 2:** Throughput, jitter and packet loss for different models.

Network type	Mark	Model 1	Model 2	MPLLRM-SDN
LAN	Throughput/bps	136	243	298
	Jitter/ms	2.36	1.28	0.19
	Packet loss/%	0.018	0.009	0.002
MAN	Throughput/bps	68	124	165
	Jitter/ms	3.58	1.41	0.21
	Packet loss/%	0.026	0.017	0.004
WAN	Throughput/bps	115	183	214
	Jitter/ms	2.97	1.59	0.13
	Packet loss/%	0.015	0.006	0.001

It can be seen from the data in Table 2 that the performance of MPLRM-SDN model is superior to the other two models in different network types. In the LAN, the throughput of the MPLRM-SDN Model was 298 bps, significantly higher than the 136 bps of Model 1 and 243 bps of Model 2. In MAN, the throughput of MPLRM-SDN model is 165 bps, and in WAN, the throughput of MPLRM-SDN model is 214 bps, both higher than the other two models. The increase in throughput is mainly due to the introduction of the multipath transport strategy in the MPLRM-SDN model and the centralized management of the SDN controller, which together enable more efficient network resource utilization and faster data transfer. In terms of jitter, the MPLRM-SDN model has the lowest jitter value in all network conditions, which is 0.19 ms in local area network, 0.21 ms in metropolitan area network, and 0.13 ms in wide area network. Low jitter indicates high stability of data transmission without large delay fluctuations, which is particularly important for applications with high real-time requirements.

The reason for the reduction of jitter is that MPLRM-SDN model adopts real-time network monitoring and dynamic resource adjustment, which can quickly respond to the change of network state and ensure the continuity and stability of data transmission. In terms of packet loss rate, the packet loss rate of MPLRM-SDN model under the three network conditions is 0.002%, 0.004% and 0.001% respectively, which is significantly lower than the other two models. A low packet loss rate indicates a higher reliability of data transmission and a lower probability of data loss. The reduction of packet loss rate is due to the network slicing technology and multi-path transmission strategy used in the MPLRM-SDN model, which can effectively allocate transmission tasks and avoid the congestion and packet loss of a single path. To sum up, the advantages of MPLRM-SDN model in computational efficiency are obviously reflected in higher throughput, lower jitter and lower packet loss rate. These advantages make the model show excellent computing performance in various network environments.

**Fig 10:**
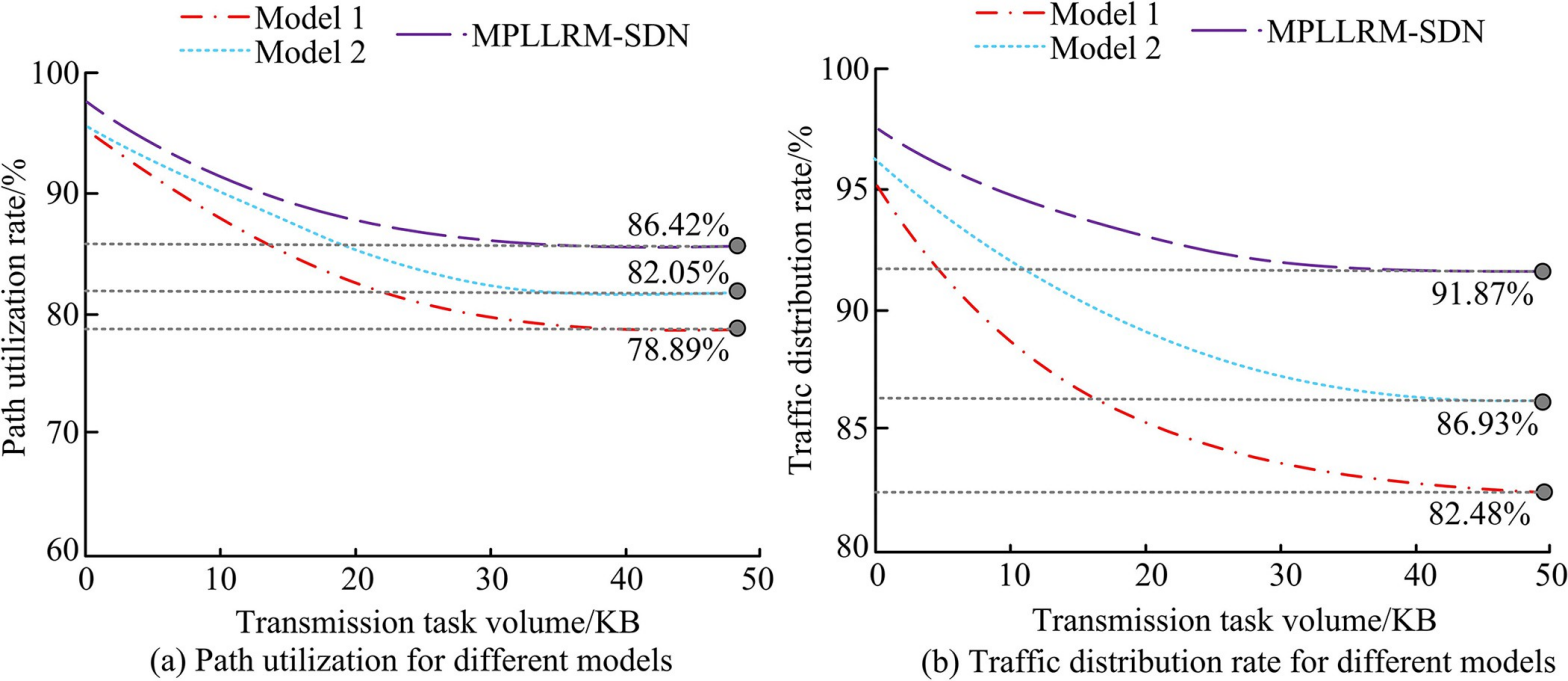
Load balancing performance of different models.

Path utilization rate and traffic distribution rate are selected as the indicators to measure the load balancing of the three models, and the load balancing performance of the three models is obtained as shown in Fig 10. In Fig 10A, the path utilization of Model 1, Model 2, and Model MPLLRM-SDN all show a decreasing trend as the transmission task volume increases. When the transmission task volume reaches 50KB, the path utilization rates of model MPLLRM-SDN, model 2, and model 1 are 86.42%, 82.05%, and 78.89%, respectively. In Fig 10B, after the transmission task volume reaches 50KB, the traffic allocation rates of model MPLLRM-SDN, model 2, and model 1 are 91.87%, 86.93%, and 82.48%, respectively.

In addition to selecting Models 1 and 2 for comparison with the proposed methods, the research also selected three recent relevant literatures for comparative analysis. The performance test results of the four methods under the same simulation environment are shown in Table 3.

**Table 3:** Performance test results of the four methods.

Model	Average latency /ms	End to end delay /ms	Throughput /bps	Jitter /ms	Packet loss rate /%	References
p-SDN	28.94	39.17	298	0.65	0.016	Reference [34]
SDCRL	22.57	33.26	310	0.38	0.008	Reference [35]
MFO-SDN- ec	30.06	37.48	306	0.72	0.012	Reference [36]
MPLLRM-SDN	21.32	31.25	326	0.15	0.001	This study

The results of the performance tests conducted on the four methods under identical simulation conditions are presented in Table 3. As illustrated in Table 3, the proposed method exhibits superior performance compared to the other three most recent methods. The average latency and end-to-end latency are 21.32 ms and 31.25 ms, respectively, while the throughput is 326 bps, the jitter is 0.15 ms, and the packet loss rate is 0.001. In conclusion, the performance of the proposed method in the simulation test is significantly superior to that of Model 1, Model 2, and the three most recent comparison methods.

### 4.2 Effectiveness analysis of MPLLRM-SDN model in practical applications

**Fig 11:**
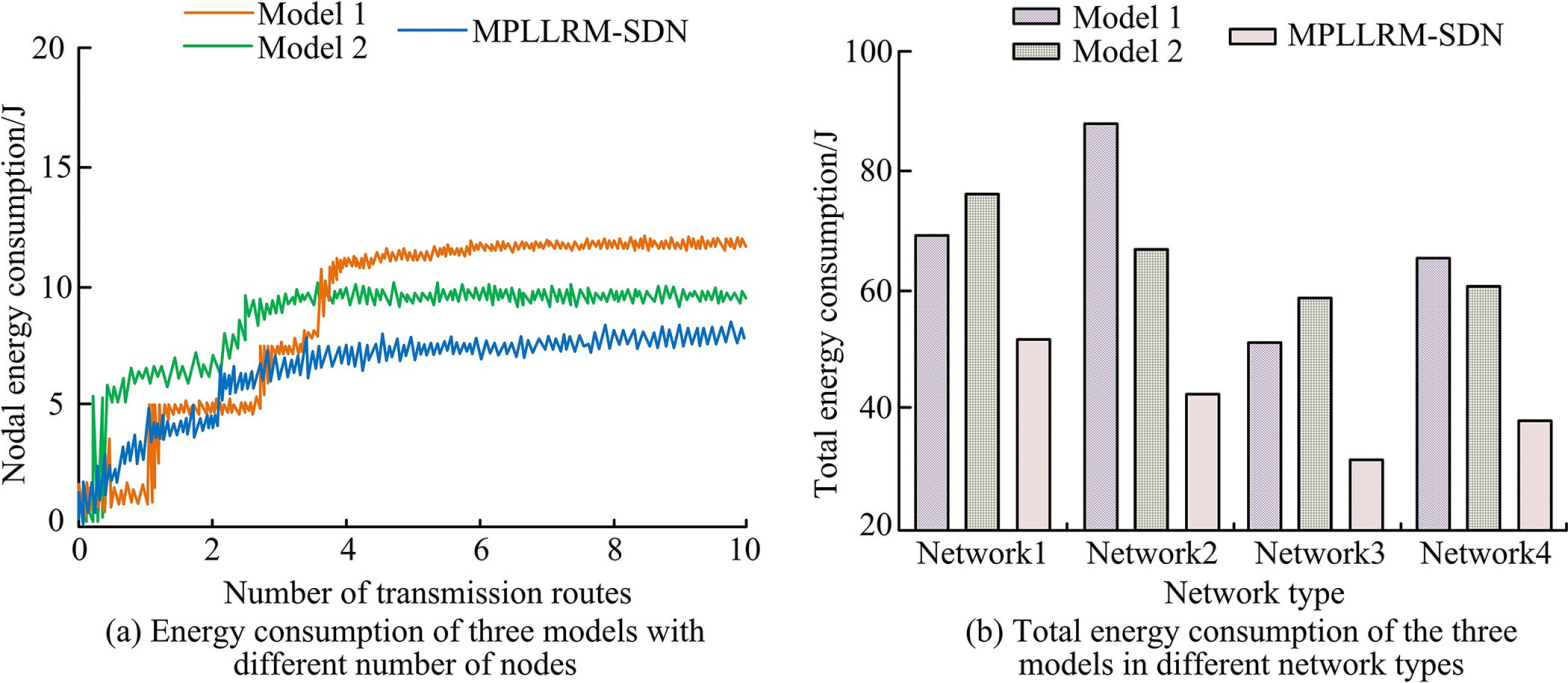
Energy performance of different models in real applications.

In addition to analyzing the performance of the three models in the simulation environment, it is also necessary to apply the three models to the real environment and test the performance of the three models in real applications. WLAN networks, cellular networks, personal area networks, and 5GCN are selected as four different network types. Moreover, the four network types are denoted as Network 1, Network 2, Network 3, and Network 4, respectively, and the energy performance of the three models under the four network types is obtained as shown in Fig 11.

**Fig 12:**
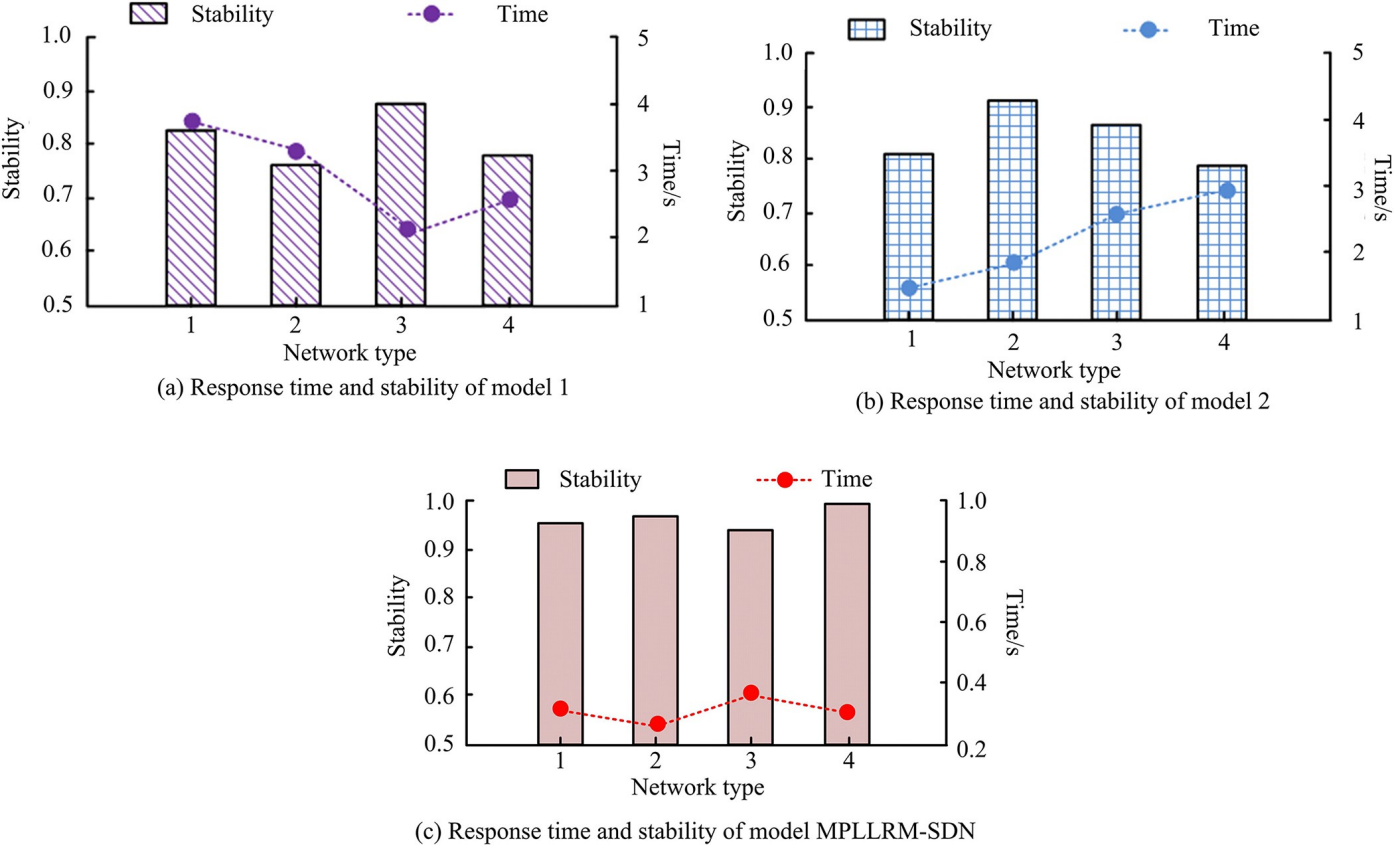
Response time and stability of different models.

Fig 11A and 11B show the node energy consumption and total network energy consumption of the three models for the four network types, respectively. In Fig 11A, when the number of transmission routes increases from 0 to 10, the node energy consumption values of the three models increase continuously and finally stabilize at one value. When the node energy consumption value stabilizes, the maximum node energy consumption values of Model 1, Model 2, and Model MPLLRM-SDN are 12.36 J, 9.89 J, and 7.12 J, respectively. In Fig 11B, the total energy consumption value of Model MPLLRM-SDN is the lowest in comparison to both Model 1 and Model 2 for the four different network types. When the network type is personal area network, the total energy consumption value of MPLLRM-SDN is the lowest, which is only 32.64 J. Further testing the practical effect of the three models, the response time and stability of the three models under the four network types are obtained as shown in Fig 12.

Fig 12 shows the response time and stability of the three models in the four network types, respectively. Model 1, Model 2, and Model MPLLRM-SDN can have the highest stability of 0.87, 0.91, and 0.98 among the four network types, and the response time spent is as high as 3.8s, 2.9s, and 0.3s, respectively. Taken together, Model MPLLRM-SDN possesses better stability and faster response time. Taking the actual sending delay and the actual TD as the test metrics, the delay performance of the three models in the four network types is obtained as shown in Table 4.

**Table 4:** Send delay and transmission delay for different models in four networks.

Network type	Delay	Model 1	Model 2	MPLLRM-SDN
Network 1	Send delay/ms	13.36	11.35	6.05
	Transmission delay/ms	33.14	28.58	13.64
Network 2	Send delay/ms	11.28	8.89	5.12
	Transmission delay/ms	35.18	24.66	19.04
Network 3	Send delay/ms	16.39	13.05	4.76
	Transmission delay/ms	39.32	28.61	20.57
Network 4	Send delay/ms	9.94	5.82	3.98
	Transmission delay/ms	41.20	36.23	15.25

Table 4 shows the actual sending delay and TD of the three models in four networks. Under the four different network types, the actual sending delay and TD of MPLLRM-SDN are smaller than those of Model 1 and Model 2. e.g., in Network 1, the sending delay and TD of Model 1 are 13.36ms and 33.14ms, respectively, the sending delay and TD of Model 2 are 11.35ms and 28.58ms, respectively, and the sending delay and TD of Model MPLLRM-SDN has a send delay and TD of 6.05ms and 13.64ms, respectively.

The efficacy and security of the proposed model MPLRM-SDN and the models referenced in [34–36] must be tested under four distinct network types: WLAN, cellular, personal area, and 5G communication. The results are shown in Table 5.

**Table 5:** Actual performance and security performance of four models in four types of networks.

Model	Average latency /ms	Total energy consumption /J	Total throughput /bps	Reliability /%	Safety score	References
p-SDN	31.26	17.78	275	97.78	8.4	Reference [34]
SDCRL	30.19	20.05	281	97.24	8.1	Reference [35]
MFO-SDN- ec	28.47	18.92	273	96.12	8.8	Reference [36]
MPLLRM-SDN	25.38	15.65	315	98.95	9.3	This study

As evidenced by the results of the actual test in Table 5, the MPLRM-SDN model demonstrates superior performance in practical applications when compared to the other three models. The average delay of the MPLRM-SDN model in the four types of networks is as low as 25.38ms, the total energy consumption is as low as 15.65J, the total throughput reaches 315bps, the reliability of the model is as high as 98.95%, and the security score of 9.3 is closer to the full score of 10. It can be concluded that the MPLRM-SDN model has higher security and better performance in practical applications.

## 5. Discussion

The MPLRM-SDN model is proposed to solve the problems of high data transmission delay, network congestion and unreasonable resource allocation in PIoT. The experimental results show that the MPLRM-SDN model performs better than the traditional single path transmission method and other existing multipath transmission methods under different network conditions. Specifically, this model shows significant advantages in key performance indicators such as throughput, jitter, and packet loss rate. In addition, by introducing the centralized management and dynamic resource adjustment mechanism of SDN controller, this model can optimize the path and assign tasks under the real-time network state, effectively reduce the data transmission delay, and improve the stability and transmission reliability of the network. Compared with other similar studies, the MPLRM-SDN model has significant advantages. For example, compared with the research of S. Bhardwaj and S. N. Panda [33], although this research shows the performance of RYU SDN controller in different network environments, its main focus is on the performance evaluation of the controller itself, which fails to fully solve the problems of multipath transmission and dynamic task allocation. By introducing dynamic task allocation mechanism and multi-path optimization strategy, this study not only improves the overall network resource utilization, but also significantly reduces the delay and jitter of data transmission, and improves the stability and reliability of transmission. In addition, compared with the research of M. Tavasoli et al. [35], this study proposed a cache, routing and load balancing algorithm based on SDN, which mainly solved the resource allocation problem in the information center network. However, the optimization effect of this method is limited in dealing with high latency and network congestion in electric iot. On the other hand, by combining network slicing technology and dynamic task allocation strategy, MPLRM-SDN model is not only suitable for power iot environment, but also shows lower transmission delay, higher throughput and lower packet loss rate in experiments, showing stronger adaptability and optimization ability.

To sum up, the MPLRM-SDN model in this study has shown significant advantages in both method innovation and practical application. Through dynamic adjustment of task assignment and optimization of transmission path, efficient and low-delay data transmission is realized. Future research could further optimize the model, improve its adaptability and performance in more complex network environments, and provide more efficient solutions for data transmission in power iot and other fields.

## 6. Conclusions

To further improve the efficiency of RT in PIoT, this research combines the SDN controller and network slicing techniques to design a multi-path, low-latency RT model under the task assignment concept, i.e., MPLLRM-SDN. In the simulation test, the average delay and end-to-end delay of Model 1, Model 2, and Model MPLLRM-SDN increased with the increase of transmission task volume. When the transmission task volume was 50KB, the average delay of the three models was 53.27ms, 40.15ms, and 23.38ms, respectively, and the end-to-end delay was 78.69ms, 64.65ms, and 36.59ms, respectively. The throughput, jitter, and packet loss of the three models were tested under LAN, MAN, and WAN, respectively. It was found that the MPLLRM-SDN in the LAN achieved the highest throughput of up to 298bps. In addition, the model was able to achieve the lowest jitter and the lowest packet loss rate in WAN, which were 0.13ms and 0.001%, respectively. Testing the load balancing of the three models, it was found that MPLLRM-SDN has the highest path utilization and traffic distribution rate, which are 86.42% and 91.87%, respectively. In real applications, the energy consumption of MPLLRM-SDN was lower than that of Models 1 and 2. When the network type was personal area network, the total energy consumption value of MPLLRM-SDN was the lowest, which was only 32.64 J. Finally, the actual sending delay and transmitting delay of the three models were tested under four network types, namely, WLAN network, cellular network, personal area network, and 5G-CN. It was found that each of the three models had the highest path utilization and traffic distribution. It was concluded that all the delay values of MPLLRM-SDN are less than the other two models. In conclusion, the MPLLRM-SDN model developed in this study exhibits reduced delay values and enhanced stability in WLAN networks, cellular networks, personal area networks, and 5G-CN. However, 5G-CN represents a relatively limited number of actual network types, and further research is required to test the performance of the model in a wider range of network types.

## 7. Future work

The MPLRM-SDN model proposed in this study has significant advantages in theory, but it still faces some difficulties in practical application. First, the implementation of this method requires large-scale deployment of SDN controllers and network slicing technology, which increases the initial cost and implementation difficulty. Second, SDN technology is highly dependent on the control channel, which may encounter the problems of control channel delay and packet loss, which affects the system performance. Finally, the requirements and constraints of different PIoT application scenarios are inconsistent, and how to flexibly adjust and optimize is also a challenge. Future research can be further explored in the following directions. First, a more efficient SDN controller deployment scheme is investigated to reduce the cost and improve the stability of the control channel. Second, more intelligent algorithms will be developed to improve the real-time and reliability of MPT strategies. Finally, automated network configuration combined with artificial intelligence and advanced machine learning techniques will be used to improve the adaptive and fault handling capabilities of the system.

## Minimal data set definition

Minimal Data Set Definition at URL:

http://datadryad.org/stash/share/aB9fkw9QQ49i3tl1AzmhZpu7VU23FNLJCybZWYPwgcw

## Abbreviations

TD: Transmission Delay
PIoT: Power Internet of Things
SDN: Software-Defined Networking
Trans-P: Transmission Path
MPLRM-SDN: Multi-Path Low-Delay Routing Optimization Transmission Model Based on SDN
IoT: Internet of Things
QoS: Quality of Service
CN: Communication Networks
CNT: Core Network Transmission
APT: Air-Port Transmission
AL: Application Layer
SFC: Service Function Chaining
PRL: Physical Resource Layer
NS: Network Slice
NFV: Network Function Virtualization
LAN: Local Area Network
MAN: Metropolitan Area Network
WAN: Wide Area Network
